# Supplementary material for: Substantial changes in Gaseous pollutants and health effects during COVID-19 in Delhi, India
Source: PeerJ. 2023 Jan 9;11:e14489. doi: 10.7717/peerj.14489 (PMC9835704; doi:10.7717/peerj.14489)
Supplement: Supplemental Information 1 [file peerj-11-14489-s001.docx]

**Supplementary**

**Substantial Changes in Gaseous Pollutants and Health Effects During The COVID-19**

Bhupendra Pratap Singh^1,2*^, Puneeta Pandey^3^, Saikh Mohammad Wabaidur^4^, Ram Avtar^5^, Shakilur Rahman^6^

*^1^Delhi School of Climate Change and Sustainability (Institute of Eminence) University of Delhi, Delhi, India*

*^2^Deshbandhu College, University of Delhi, Delhi, India*

*^3^ School of Environment and Earth Sciences, Central University of Punjab, Bathinda, Punjab, India*

*^4^* Chemistry Department, College of Science, King Saud University, Riyadh 11451, Saudi Arabia

*^5^* Faculty of Environmental Earth Science, Hokkaido University, Sapporo, Japan, 060-0810

^6^ *Department of Medical Elementology and Toxicology, School of Chemical and Life Sciences, Jamia Hamdard,*

Stable 1: Several recent studies across the world during the lockdown period.

| **The study area (country, city)** | **Key findings for gaseous pollutants** | **Author (year)** |
| --- | --- | --- |
| India (Delhi) | The values NO_2,_ NOx, and NH_3_ were reduced by 38%, 56%, and 20%, whereas the value of O_3_ was increased by 75% during the lockdown period compared to the pre-lockdown period. | Present Study |
| Spain (Valencia, Madrid, Barcelona, Bilbao, Sevilla) | The average PM_10_ and NO_2_ declined by 70% to 80% in different Spain cities, respectively. | Carrasco et al., (2021) |
| India (Delhi) | The NO_2_, CO, and VOC declined by 50%, 37%, 38%, respectively, during the lockdown period of 2020 as compared to a similar period in 2019. The | Rathod et al., (2021) |
| France (Lyon) | The average concentration of NO_2_, NO, and CO levels were reduced by 67%, 78%, and 62%, respectively. However, during the lockdown, O3, PM10, and PM2.5 were increased by 105%, 23%, and 53%, respectively. | Sbai et al., (2021) |
| Saudi Arabia (Makkah) | Compared with the pre-pandemic period, a significant decrease of concentration rates during the lockdown period, by 26.34% for SO2, 28.99% for NO_2_, 26.24% for CO, 11.62% for O_3_, and 30.03% for PM_10_. | Morsy et al., (2021) |
| China | An increase of 37.8%, 32.3%, and 14.2% in the number of COVID-19 cases for every 10-mg/m3 increase in long-term exposure to NO_2_, PM_2.5_, and PM_10_, respectively. | Zheng et al., (2021) |
| United Kingdom (South East, UK) | NO_2_ concentrations decreased by an average of 14–38%, compared to the mean of the same period over the preceding 5-years. | Wyche et al., (2021) |
| United State of America | During stay-at-home orders, average PM2.5 levels were slightly higher (~10%) than expected; average) O_3_, NO_2_, CO, and PM_10_ levels were slightly lower (~30%, ~20%, ~27%, and ~1% of their IQR, respectively) than expected. | Bekbulat et. al., (2021) |
| Chile (Santiago) | Significant concentrations in Chile were reduced by 54%, 13%, and 11% for NOx, CO, and PM_2.5_, respectively, during the pandemic period compared to the historical period. | Toro et al., (2021) |
| Malaysia (Kota Kinabalu, Kuantan, Alor Star, Kota Bharu, and Ipoh) | Significant reductions of PM_10_, PM_2.5_, SO_2_, O_3,_ and CO were recorded at Kota Kinabalu (17%), Kuantan (9.5%), Alor Star (38%), Kota Bharu (15%), and Ipoh (27%), respectively. | Othman & Latif (2021) |
| Italy (Milan) | A significant increase of ground-level daily average O_3_ concentrations by a factor of 2.25, while daily average NO_2_ concentrations exhibited decreased levels by 64.7%. | Zoran et al., (2020) |
| Jordan (Irbid) | The concentrations were decline by various percentages (from 29% for PM_10_ to 72% for NO_2_) as compared to their concentration before the pandemic period. | Shatnawi et al., (2020) |
| Korea | The average levels of PM_2.5_, PM_10_, NO_2_, and CO decreased by 45.45%, 35.56%, 20.41%, and 17.33%, respectively. | Ju et al., (2020) |
| United Kingdom | Measurements from 129 monitoring stations identified mean reductions in NO_2_ of 38.3% and PM_2.5_ of 16.5%, respectively, during the lockdown period. | Jephcote et al., (2020) |

Stable 2: Distribution of monitoring station in various zones in Delhi

| District | Zone |
| --- | --- |
| East Delhi | Arya Nagar, Vivek Vihar, Okhla, Patparganj |
| New Delhi | R.K. Puram |
| North Delhi | Alipur, Narela, Wazirpur. North Campus, Jahangirpuri |
| North East Delhi | ITO |
| North West Delhi | Rohini, Ashok Vihar , DTU |
| South Delhi | Karni Singh, Lodhi Garden, JLN Stadium, Aurobindo Marg |
| South East Delhi | Okhla, Siri fort |
| South West Delhi | Najafgarh, Sri Aurobindo Marg |

(<https://ceodelhi.gov.in/OnlineErms/Reports/PS_LocationListOn15thOctDistrictwise.aspx>)

Stable 3: Hazard quotients of NO_2_, O_3_, and SO_2_ for daily and annual exposure groups.

|  | East Delhi | | | | | | | | New Delhi | | | | | | | | North Delhi | | | | | | | North East Delhi | | | | | | | North West Delhi | | | | | | South Delhi | | | | | | | South East Delhi | | | | | South West Delhi | | | | | | | | |  |
| --- | --- | --- | --- | --- | --- | --- | --- | --- | --- | --- | --- | --- | --- | --- | --- | --- | --- | --- | --- | --- | --- | --- | --- | --- | --- | --- | --- | --- | --- | --- | --- | --- | --- | --- | --- | --- | --- | --- | --- | --- | --- | --- | --- | --- | --- | --- | --- | --- | --- | --- | --- | --- | --- | --- | --- | --- | --- | --- |
|  | Infant (0-1 yrs) | | Toddler (2-5 yrs) | | | Child (6-12 yrs) | | Adult (19-75 yrs) | Infant (0-1 yrs) | Toddler (2-5 yrs) | | | Child (6-12 yrs) | | Adult (19-75 yrs) | | Infant (0-1 yrs) | Toddler (2-5 yrs) | | | Child (6-12 yrs) | | Adult (19-75 yrs) | Infant (0-1 yrs) | Toddler (2-5 yrs) | | | Child (6-12 yrs) | | Adult (19-75 yrs) | Infant (0-1 yrs) | Toddler (2-5 yrs) | | Child (6-12 yrs) | | Adult (19-75 yrs) | Infant (0-1 yrs) | | Toddler (2-5 yrs) | | Child (6-12 yrs) | | Adult (19-75 yrs) | Infant (0-1 yrs) | | Child (2-5 yrs) | | Child (6-12 yrs) | Adult (19-75 yrs) | Infant (0-1 yrs) | | Child (2-5 yrs) | | Child (6-12 yrs) | | Adult (19-75 yrs) | |  |
| NO_2_ | 0.72 | | 0.66 | | | 0.41 | | 0.26 | 0.87 | 0.80 | | | 0.50 | | 0.31 | | 0.84 | 0.76 | | | 0.48 | | 0.30 | 0.72 | 0.54 | | | 0.34 | | 0.21 | 0.72 | 0.69 | | 0.43 | | 0.27 | 0.76 | | 0.69 | | 0.43 | | 0.27 | 0.96 | | 0.87 | | 0.55 | 0.35 | 0.47 | | 0.43 | | 0.27 | | 0.17 | |  |
| O_3_ | 0.17 | | 0.16 | | | 0.10 | | 0.06 | 0.16 | 0.14 | | | 0.09 | | 0.06 | | 0.15 | 0.14 | | | 0.08 | | 0.05 | 0.17 | 0.12 | | | 0.08 | | 0.05 | 0.17 | 0.25 | | 0.16 | | 0.10 | 0.26 | | 0.24 | | 0.15 | | 0.09 | 0.26 | | 0.24 | | 0.15 | 0.09 | 0.30 | | 0.28 | | 0.17 | | 0.11 | |  |
| SO_2_ | NA | | NA | | | NA | | NA | NA | NA | | | NA | | NA | | NA | NA | | | NA | | NA | NA | 0.04 | | | 0.14 | | 0.09 | NA | 0.05 | | 0.18 | | 0.11 | 0.23 | | 0.04 | | 0.13 | | 0.08 | 0.19 | | 0.03 | | 0.11 | 0.07 | 0.14 | | 0.02 | | 0.08 | | 0.05 | |  |
|  | East Delhi | | | | | | | | New Delhi | | | | | | | | North Delhi | | | | | | | North East Delhi | | | | | | | North West Delhi | | | | | | South Delhi | | | | | | | South East Delhi | | | | | | | South West Delhi | | | | | | |  |
|  | | Infant (0-1 yrs) | | Child (2-5 yrs) | Child (6-12 yrs) | | Adult (19-75 yrs) | | Infant (0-1 yrs) | | Child (2-5 yrs) | Child (6-12 yrs) | | Adult (19-75 yrs) | | Infant (0-1 yrs) | | | Child (2-5 yrs) | Child (6-12 yrs) | | Adult (19-75 yrs) | | Infant (0-1 yrs) | | Child (2-5 yrs) | Child (6-12 yrs) | | Adult (19-75 yrs) | | Infant (0-1 yrs) | Child (2-5 yrs) | Child (6-12 yrs) | | Adult (19-75 yrs) | | Infant (0-1 yrs) | Child (2-5 yrs) | | Child (6-12 yrs) | | Adult (19-75 yrs) | | Infant (0-1 yrs) | Child (2-5 yrs) | | Child (6-12 yrs) | | Adult (19-75 yrs) | | Infant (0-1 yrs) | | Child (2-5 yrs) | | Child (6-12 yrs) | | Adult (19-75 yrs) | |
| NO_2_ | | 0.51 | | 0.39 | 0.24 | | 0.15 | | 0.58 | | 0.53 | 0.33 | | 0.21 | | 0.42 | | | 0.38 | 0.24 | | 0.15 | | 0.62 | | 0.57 | 0.35 | | 0.22 | | 0.75 | 0.38 | 0.24 | | 0.15 | | 0.76 | 0.36 | | 0.22 | | 0.14 | | 0.39 | 0.36 | | 0.22 | | 0.14 | | 0.27 | | 0.25 | | 0.16 | | 0.10 | |
| O_3_ | | NA | | 0.21 | 0.13 | | 0.08 | | 0.22 | | 0.20 | 0.13 | | 0.08 | | 0.20 | | | 0.18 | 0.11 | | 0.07 | | 0.20 | | 0.19 | 0.12 | | 0.07 | | 0.27 | 0.38 | 0.24 | | 0.15 | | 0.26 | 0.29 | | 0.18 | | 0.12 | | 0.23 | 0.21 | | 0.13 | | 0.08 | | 0.18 | | 0.17 | | 0.10 | | 0.07 | |
| SO_2_ | | NA | | 0.06 | 0.20 | | 0.13 | | NA | | NA | NA | | NA | | 0.23 | | | 0.04 | 0.13 | | 0.08 | | 0.11 | | 0.02 | 0.06 | | 0.04 | | 0.32 | 0.08 | 0.29 | | 0.18 | | 0.23 | 0.03 | | 0.10 | | 0.06 | | 0.15 | 0.03 | | 0.09 | | 0.05 | | 0.10 | | 0.02 | | 0.06 | | 0.04 | |

Stable 4: Correlation of NO with various sites at Delhi

| **Correlations NO** | | | | | | | | | | | | | | | | | | | |
| --- | --- | --- | --- | --- | --- | --- | --- | --- | --- | --- | --- | --- | --- | --- | --- | --- | --- | --- | --- |
|  | Alipur | | Arya Nagar | Ashok Vihar | Bawana | DTU | ITO | JLN | Karni Singh | Lodhi Road | Najafgarh | Okhla | Patparjanj | R K Puram | Rohini | Sirifort | Sri Aurobindo Marge | Vivek Vihar | Wazirpur |
| Alipur | 1.00 | |  |  |  |  |  |  |  |  |  |  |  |  |  |  |  |  |  |
| Arya Nagar | 0.12 | | 1.00 |  |  |  |  |  |  |  |  |  |  |  |  |  |  |  |  |
| Ashok Vihar | .499^**^ | | -0.03 | 1.00 |  |  |  |  |  |  |  |  |  |  |  |  |  |  |  |
| Bawana | .798^**^ | | 0.08 | .507^**^ | 1.00 |  |  |  |  |  |  |  |  |  |  |  |  |  |  |
| DTU | .670^**^ | | 0.05 | .629^**^ | .770^**^ | 1.00 |  |  |  |  |  |  |  |  |  |  |  |  |  |
| ITO | .484^**^ | | -0.03 | .245^**^ | .584^**^ | .302^**^ | 1.00 |  |  |  |  |  |  |  |  |  |  |  |  |
| JLN | .815^**^ | | 0.05 | .559^**^ | .836^**^ | .638^**^ | .594^**^ | 1.00 |  |  |  |  |  |  |  |  |  |  |  |
| Karni Singh | .714^**^ | | 0.06 | .500^**^ | .807^**^ | .667^**^ | .509^**^ | .814^**^ | 1.00 |  |  |  |  |  |  |  |  |  |  |
| Lodhi Road | -0.04 | | -0.04 | -.127^*^ | -0.02 | -0.07 | 0.05 | -0.05 | -0.06 | 1.00 |  |  |  |  |  |  |  |  |  |
| Najafgarh | .662^**^ | | 0.11 | .401^**^ | .784^**^ | .761^**^ | .367^**^ | .630^**^ | .750^**^ | -0.09 | 1.00 |  |  |  |  |  |  |  |  |
| Okhla | -0.06 | | 0.15 | -.161^*^ | -0.10 | -0.13 | -.221^**^ | -0.11 | -0.08 | -0.09 | -0.08 | 1.00 |  |  |  |  |  |  |  |
| Patparjanj | .805^**^ | | 0.07 | .533^**^ | .938^**^ | .783^**^ | .591^**^ | .866^**^ | .799^**^ | 0.00 | .775^**^ | -0.08 | 1.00 |  |  |  |  |  |  |
| R K Puram | .744^**^ | | 0.08 | .413^**^ | .838^**^ | .670^**^ | .587^**^ | .813^**^ | .765^**^ | -0.08 | .770^**^ | -0.08 | .822^**^ | 1.00 |  |  |  |  |  |
| Rohini | .714^**^ | | 0.06 | .500^**^ | .807^**^ | .667^**^ | .509^**^ | .814^**^ | 1.000^**^ | -0.06 | .750^**^ | -0.08 | .799^**^ | .765^**^ | 1.00 |  |  |  |  |
| Sirifort | .283^**^ | | -0.02 | .393^**^ | .427^**^ | .454^**^ | .414^**^ | .401^**^ | .511^**^ | -0.02 | .406^**^ | -0.02 | .474^**^ | .345^**^ | .511^**^ | 1.00 |  |  |  |
| Sri Aurobindo Marge | 0.10 | | 0.00 | .282^**^ | 0.02 | .282^**^ | -.249^**^ | 0.02 | .183^**^ | -.212^**^ | .215^**^ | -0.10 | 0.01 | 0.06 | .183^**^ | .200^**^ | 1.00 |  |  |
| Vivek Vihar | .413^**^ | | 0.03 | .347^**^ | .511^**^ | .412^**^ | .722^**^ | .531^**^ | .454^**^ | 0.05 | .267^**^ | -0.12 | .549^**^ | .532^**^ | .454^**^ | .408^**^ | -0.06 | 1.00 |  |
| Wazirpur | .259^**^ | | -0.02 | .348^**^ | .352^**^ | .389^**^ | .471^**^ | .386^**^ | .440^**^ | -.159^*^ | .286^**^ | -0.11 | .325^**^ | .364^**^ | .440^**^ | .697^**^ | .293^**^ | .475^**^ | 1.00 |
| **. Correlation is significant at the 0.01 level (2-tailed). | | | | | | | | | | | | | | | | | | |  |
| *. Correlation is significant at the 0.05 level (2-tailed). | | | | | | | | | | | | | | | | | | |  |

Stable 5: Correlation of NO_2_ with various sites at Delhi

| **Correlations NO_2_** | | | | | | | | | | | | | | | | | |  |
| --- | --- | --- | --- | --- | --- | --- | --- | --- | --- | --- | --- | --- | --- | --- | --- | --- | --- | --- |
|  | Alipur | Arya Nagar | Ashok Vihar | Bawana | DTU | ITO | JLN | Karni Singh | Najafgarh | Okhla | Patparganj | R K Puram | Rohini | Sirifort | Sri Aurobindo Marge | Vivek Vihar | Wazirpur |  |
| Alipur | 1 |  |  |  |  |  |  |  |  |  |  |  |  |  |  |  |  |  |
| Arya Nagar | -.375^**^ | 1 |  |  |  |  |  |  |  |  |  |  |  |  |  |  |  |  |
| Ashok Vihar | .545^**^ | -.216^**^ | 1 |  |  |  |  |  |  |  |  |  |  |  |  |  |  |  |
| Bawana | .642^**^ | -.266^**^ | .579^**^ | 1 |  |  |  |  |  |  |  |  |  |  |  |  |  |  |
| DTU | .527^**^ | -.320^**^ | .550^**^ | .760^**^ | 1 |  |  |  |  |  |  |  |  |  |  |  |  |  |
| ITO | -.124^*^ | .329^**^ | .198^**^ | .127^*^ | 0.003 | 1 |  |  |  |  |  |  |  |  |  |  |  |  |
| JLN | .652^**^ | -.408^**^ | .596^**^ | .848^**^ | .762^**^ | 0.016 | 1 |  |  |  |  |  |  |  |  |  |  |  |
| Karni Singh | .444^**^ | -0.096 | .483^**^ | .741^**^ | .613^**^ | 0.102 | .671^**^ | 1 |  |  |  |  |  |  |  |  |  |  |
| Najafgarh | .682^**^ | -.301^**^ | .569^**^ | .786^**^ | .662^**^ | 0.022 | .775^**^ | .489^**^ | 1 |  |  |  |  |  |  |  |  |  |
| Okhla | .543^**^ | -.304^**^ | .600^**^ | .859^**^ | .774^**^ | .138^*^ | .880^**^ | .752^**^ | .724^**^ | 1 |  |  |  |  |  |  |  |  |
| Patparganj | .656^**^ | -.338^**^ | .503^**^ | .826^**^ | .671^**^ | 0.013 | .786^**^ | .656^**^ | .689^**^ | .802^**^ | 1 |  |  |  |  |  |  |  |
| R K Puram | .392^**^ | -0.041 | .386^**^ | .647^**^ | .533^**^ | .179^**^ | .639^**^ | .663^**^ | .498^**^ | .708^**^ | .621^**^ | 1 |  |  |  |  |  |  |
| Rohini | .444^**^ | -0.099 | .484^**^ | .746^**^ | .617^**^ | 0.105 | .671^**^ | 1.000^**^ | .489^**^ | .753^**^ | .656^**^ | .659^**^ | 1 |  |  |  |  |  |
| Sirifort | .494^**^ | -.291^**^ | .598^**^ | .734^**^ | .711^**^ | 0.065 | .758^**^ | .471^**^ | .792^**^ | .798^**^ | .629^**^ | .482^**^ | .481^**^ | 1 |  |  |  |  |
| Sri Aurobindo Marge | 0.040 | -.670^**^ | .180^**^ | 0.115 | .248^**^ | -.131^*^ | .286^**^ | 0.125 | 0.121 | .256^**^ | .184^**^ | 0.016 | 0.125 | .214^**^ | 1 |  |  |  |
| Vivek Vihar | .563^**^ | -.255^**^ | .461^**^ | .702^**^ | .600^**^ | -0.020 | .748^**^ | .474^**^ | .722^**^ | .676^**^ | .629^**^ | .585^**^ | .481^**^ | .671^**^ | 0.119 | 1 |  |  |
| Wazirpur | .636^**^ | -.204^**^ | .555^**^ | .775^**^ | .599^**^ | -0.010 | .709^**^ | .559^**^ | .756^**^ | .669^**^ | .720^**^ | .533^**^ | .569^**^ | .709^**^ | -0.055 | .745^**^ | 1 |  |
| **. Correlation is significant at the 0.01 level (2-tailed). | | | | | | | | | | | | | | | | | | |
| *. Correlation is significant at the 0.05 level (2-tailed). | | | | | | | | | | | | | | | | | | |

Stable 6: Correlation of NOx with various sites at Delhi

| **Correlations NOx** | | | | | | | | | | | | | | | | | |  |
| --- | --- | --- | --- | --- | --- | --- | --- | --- | --- | --- | --- | --- | --- | --- | --- | --- | --- | --- |
|  | Alipur | Arya Nagar | Ashok Vihar | Bawana | DTU | ITO | JLN | Karni Singh | Najafgarh | Okhla | Patparganj | R K Puram | Rohini | Sirifort | Sri Aurobindo Marge | Vivek Vihar | Wazirpur |  |
| Alipur | 1 |  |  |  |  |  |  |  |  |  |  |  |  |  |  |  |  |  |
| Arya Nagar | -.197^**^ | 1 |  |  |  |  |  |  |  |  |  |  |  |  |  |  |  |  |
| Ashok Vihar | .690^**^ | -.172^**^ | 1 |  |  |  |  |  |  |  |  |  |  |  |  |  |  |  |
| Bawana | .603^**^ | -0.107 | .616^**^ | 1 |  |  |  |  |  |  |  |  |  |  |  |  |  |  |
| DTU | .491^**^ | -.162^**^ | .500^**^ | .727^**^ | 1 |  |  |  |  |  |  |  |  |  |  |  |  |  |
| ITO | .528^**^ | 0.081 | .369^**^ | .715^**^ | .433^**^ | 1 |  |  |  |  |  |  |  |  |  |  |  |  |
| JLN | .664^**^ | -.206^**^ | .654^**^ | .911^**^ | .735^**^ | .645^**^ | 1 |  |  |  |  |  |  |  |  |  |  |  |
| Karni Singh | .544^**^ | -0.069 | .589^**^ | .791^**^ | .681^**^ | .415^**^ | .821^**^ | 1 |  |  |  |  |  |  |  |  |  |  |
| Najafgarh | .633^**^ | -0.097 | .532^**^ | .849^**^ | .616^**^ | .713^**^ | .805^**^ | .707^**^ | 1 |  |  |  |  |  |  |  |  |  |
| Okhla | .619^**^ | -0.097 | .635^**^ | .902^**^ | .714^**^ | .645^**^ | .938^**^ | .838^**^ | .837^**^ | 1 |  |  |  |  |  |  |  |  |
| Patparganj | .639^**^ | -.175^**^ | .632^**^ | .919^**^ | .742^**^ | .622^**^ | .919^**^ | .768^**^ | .789^**^ | .907^**^ | 1 |  |  |  |  |  |  |  |
| R K Puram | .559^**^ | -0.018 | .460^**^ | .781^**^ | .599^**^ | .599^**^ | .762^**^ | .721^**^ | .713^**^ | .740^**^ | .741^**^ | 1 |  |  |  |  |  |  |
| Rohini | .544^**^ | -0.069 | .589^**^ | .791^**^ | .681^**^ | .415^**^ | .821^**^ | 1.000^**^ | .707^**^ | .838^**^ | .768^**^ | .721^**^ | 1 |  |  |  |  |  |
| Sirifort | .550^**^ | -0.090 | .610^**^ | .874^**^ | .690^**^ | .624^**^ | .912^**^ | .770^**^ | .826^**^ | .955^**^ | .890^**^ | .725^**^ | .770^**^ | 1 |  |  |  |  |
| Sri Aurobindo Marge | .175^**^ | -.414^**^ | .371^**^ | .359^**^ | .388^**^ | 0.054 | .426^**^ | .357^**^ | .275^**^ | .406^**^ | .395^**^ | .179^**^ | .357^**^ | .404^**^ | 1 |  |  |  |
| Vivek Vihar | .469^**^ | -0.003 | .285^**^ | .489^**^ | .470^**^ | .419^**^ | .482^**^ | .422^**^ | .487^**^ | .421^**^ | .419^**^ | .516^**^ | .422^**^ | .350^**^ | 0.048 | 1 |  |  |
| Wazirpur | .467^**^ | .210^**^ | .225^**^ | .366^**^ | .345^**^ | .377^**^ | .302^**^ | .354^**^ | .412^**^ | .304^**^ | .326^**^ | .415^**^ | .354^**^ | .249^**^ | -.260^**^ | .534^**^ | 1 |  |
| **. Correlation is significant at the 0.01 level (2-tailed). | | | | | | | | | | | | | | | | | | |
| *. Correlation is significant at the 0.05 level (2-tailed). | | | | | | | | | | | | | | | | | | |

Stable 7: Correlation of O_3_ with various sites at Delhi

| **Correlations O_3_** | | | | | | | | | | | | | | |  |
| --- | --- | --- | --- | --- | --- | --- | --- | --- | --- | --- | --- | --- | --- | --- | --- |
|  | Arya Nagar | Ashok Vihar | Bawana | DTU | ITO | JLN | Karni Singh | Najafgarh | Patparganj | R K Puram | Rohini | Sri Aurobindo Marge | Vivek Vihar | Wazirpur |  |
| Arya Nagar | 1 |  |  |  |  |  |  |  |  |  |  |  |  |  |  |
| Ashok Vihar | -.306^**^ | 1 |  |  |  |  |  |  |  |  |  |  |  |  |  |
| Bawana | -.304^**^ | .542^**^ | 1 |  |  |  |  |  |  |  |  |  |  |  |  |
| DTU | -.306^**^ | .400^**^ | .540^**^ | 1 |  |  |  |  |  |  |  |  |  |  |  |
| ITO | -.334^**^ | .449^**^ | .518^**^ | .642^**^ | 1 |  |  |  |  |  |  |  |  |  |  |
| JLN | -.298^**^ | .473^**^ | .710^**^ | .737^**^ | .544^**^ | 1 |  |  |  |  |  |  |  |  |  |
| Karni Singh | -.220^**^ | .574^**^ | .771^**^ | .696^**^ | .635^**^ | .735^**^ | 1 |  |  |  |  |  |  |  |  |
| Najafgarh | .501^**^ | -0.054 | -0.099 | .226^**^ | .194^**^ | .156^*^ | .167^**^ | 1 |  |  |  |  |  |  |  |
| Patparganj | -.222^**^ | .464^**^ | .566^**^ | .451^**^ | .558^**^ | .467^**^ | .698^**^ | .129^*^ | 1 |  |  |  |  |  |  |
| R K Puram | -.377^**^ | .264^**^ | .398^**^ | .465^**^ | .503^**^ | .508^**^ | .390^**^ | .128^*^ | .270^**^ | 1 |  |  |  |  |  |
| Rohini | -.220^**^ | .574^**^ | .771^**^ | .696^**^ | .635^**^ | .735^**^ | 1.000^**^ | .167^**^ | .698^**^ | .390^**^ | 1 |  |  |  |  |
| Sri Aurobindo Marg | .214^**^ | -.185^**^ | -.246^**^ | .179^**^ | 0.096 | .212^**^ | -0.022 | .616^**^ | -0.059 | .360^**^ | -0.022 | 1 |  |  |  |
| Vivek Vihar | -.243^**^ | .497^**^ | .626^**^ | .561^**^ | .480^**^ | .681^**^ | .709^**^ | .141^*^ | .711^**^ | .425^**^ | .709^**^ | 0.091 | 1 |  |  |
| Wazirpur | -.475^**^ | .496^**^ | .629^**^ | .618^**^ | .565^**^ | .616^**^ | .623^**^ | 0.021 | .392^**^ | .374^**^ | .623^**^ | -0.023 | .504^**^ | 1 |  |
| **. Correlation is significant at the 0.01 level (2-tailed). | | | | | | | | | | | | | | | |
| *. Correlation is significant at the 0.05 level (2-tailed). | | | | | | | | | | | | | | | |

Stable 8: Correlation of NH_3_ with various sites at Delhi

| **Correlations NH_3_** | | | | | | | | | | | | | | | | | | | |
| --- | --- | --- | --- | --- | --- | --- | --- | --- | --- | --- | --- | --- | --- | --- | --- | --- | --- | --- | --- |
|  | Alipur | Arya Nagar | Ashok Vihar | Bawana | DTU | ITO | JLN | Karni Singh | Lodhi Road | Narela | North Campus | Okhla | Patparganj | R K Puram | Rohini | Sirifort | Sri Aurobindo Marge | Vivek Vihar | Wazirpur |
| Alipur | 1 |  |  |  |  |  |  |  |  |  |  |  |  |  |  |  |  |  |  |
| Arya Nagar |  |  |  |  |  |  |  |  |  |  |  |  |  |  |  |  |  |  |  |
| Ashok Vihar | -0.089 | .^a^ | 1 |  |  |  |  |  |  |  |  |  |  |  |  |  |  |  |  |
| Bawana | .204^**^ | .^a^ | 0.091 | 1 |  |  |  |  |  |  |  |  |  |  |  |  |  |  |  |
| DTU | -0.036 | .^a^ | .166^**^ | .452^**^ | 1 |  |  |  |  |  |  |  |  |  |  |  |  |  |  |
| ITO | 0.119 | .^a^ | .194^**^ | .292^**^ | .247^**^ | 1 |  |  |  |  |  |  |  |  |  |  |  |  |  |
| JLN | .168^**^ | .^a^ | .354^**^ | .637^**^ | .456^**^ | .272^**^ | 1 |  |  |  |  |  |  |  |  |  |  |  |  |
| Karni Singh | -.171^**^ | .^a^ | -0.023 | .291^**^ | -0.053 | -.344^**^ | 0.017 | 1 |  |  |  |  |  |  |  |  |  |  |  |
| Lodhi Road |  |  |  |  |  |  |  |  |  |  |  |  |  |  |  |  |  |  |  |
| Narela |  |  |  |  |  |  |  |  |  |  |  |  |  |  |  |  |  |  |  |
| North Campus |  |  |  |  |  |  |  |  |  |  |  |  |  |  |  |  |  |  |  |
| Okhla | .168^**^ | .^a^ | -0.052 | .255^**^ | 0.109 | -0.09 | 0.098 | -0.001 | .^a^ | .^a^ | .^a^ | 1 |  |  |  |  |  |  |  |
| Patparganj | -0.008 | .^a^ | .148^*^ | .630^**^ | .461^**^ | 0.093 | .580^**^ | .145^*^ | .^a^ | .^a^ | .^a^ | .241^**^ | 1 |  |  |  |  |  |  |
| R K Puram | .184^**^ | .^a^ | -.130^*^ | 0.118 | -.152^*^ | -.419^**^ | 0.048 | .311^**^ | .^a^ | .^a^ | .^a^ | 0.017 | 0.029 | 1 |  |  |  |  |  |
| Rohini | -0.086 | .^a^ | .169^**^ | .333^**^ | .289^**^ | .279^**^ | .206^**^ | .446^**^ | .^a^ | .^a^ | .^a^ | -0.088 | .252^**^ | -.203^**^ | 1 |  |  |  |  |
| Sirifort | .345^**^ | .^a^ | -.137^*^ | .461^**^ | .364^**^ | .227^**^ | .319^**^ | -.301^**^ | .^a^ | .^a^ | .^a^ | .176^**^ | .359^**^ | .121^*^ | -.127^*^ | 1 |  |  |  |
| Sri Aurobindo Marge | -0.118 | .^a^ | .156^*^ | 0.124 | -0.002 | -.252^**^ | .141^*^ | .385^**^ | .^a^ | .^a^ | .^a^ | .228^**^ | .216^**^ | -0.011 | .135^*^ | -.196^**^ | 1 |  |  |
| Vivek Vihar | .141^*^ | .^a^ | .187^**^ | .822^**^ | .388^**^ | .187^**^ | .615^**^ | .287^**^ | .^a^ | .^a^ | .^a^ | .155^*^ | .608^**^ | .131^*^ | .366^**^ | .365^**^ | .252^**^ | 1 |  |
| Wazirpur | .124^*^ | .^a^ | .186^**^ | .622^**^ | .529^**^ | .199^**^ | .542^**^ | -0.03 | .^a^ | .^a^ | .^a^ | .281^**^ | .524^**^ | .124^*^ | 0.111 | .547^**^ | 0.007 | .535^**^ | 1 |
| **. Correlation is significant at the 0.01 level (2-tailed). | | | | | | | | | | | | | | | | | | | |
| *. Correlation is significant at the 0.05 level (2-tailed). | | | | | | | | | | | | | | | | | | | |

| Stable 9: Correlation Matrix with various gaseous pollutants at Delhi | | | | | | | |  |
| --- | --- | --- | --- | --- | --- | --- | --- | --- |
|  | | NO | NO_2_ | NO_X_ | O_3_ | NH_3_ | SO_2_ |  |
| Correlation | NO | 1.000 |  |  |  |  |  |  |
|  | NO_2_ | .732 | 1.000 |  |  |  |  |  |
|  | NO_X_ | **.855** | **.815** | 1.000 |  |  |  |  |
|  | O_3_ | -.446 | -.383 | -.423 | 1.000 |  |  |  |
|  | NH_3_ | .186 | .052 | .106 | -.076 | 1.000 |  |  |
|  | SO_2_ | .041 | -.080 | .169 | -.039 | .313 | 1.000 |  |
| **. Correlation is significant at the 0.01 level (2-tailed). | | | | | | | | |
| *. Correlation is significant at the 0.05 level (2-tailed). | | | | | | | | |
